# Personals

**Published:** 1916-02

**Authors:** 


### Personals.

Dr. E. A. Hasskerl, of Chicago, will locate in Brenham permanently.

Dr. and Mrs. Fussel, of Cisco, left recently for their new home in El Paso.

Dr. Ehlinger, of Bryan, was slightly injured in an automobile wreck recently.

Dr. E. S. Adams is at home again after an extended visit in North Carolina.

Dr. T. H. McGregor, of Denison, is confined to his room as the result of a fall.

Dr. A. C. Cates and Miss Mollie Maurice Truly, of Petrolia, were married recently.

Dr. J. D. Moorman, of Pleasant Valley, has been suffering from an attack of la grippe.

Dr. B. M. Jones and family have arrived from their former home at Rhome, Texas, and are now domiciled in the home recently bought of Dr. Rochell, Burkburnett, Texas.

Dr. W. B. Pope, of McKinney, was slightly injured in an automobile wreck a few weeks ago.

Dr. C. M. Rayburn, of Winnsboro, and Miss Virginia Suit, of Temple, were recently married.

Dr. R. A. Maddox, of Abilene, had his arm painfully injured by the explosion of his shotgun.

Dr. W. O. Warren, of Pecan Gap, had his eye painfully injured by the explosion of a fire cracker.

Dr. and Mrs. Millara A. Jenkins, of Abilene, are greatly improved after an attack of la grippe.

Dr. J. M. Black, of Canyon, has returned from Cordell, Okla., where he has been for several weeks.

Dr. W. J. Stephens, of Jacksonville, and Miss Ruth Stoker, of Weatherford, were married January 4th.

Dr. Walter Shepherd has accepted a position as physician to the Pickering Lumber Company at Haslem.

Dr. Gause W. Covington, a prominent Fort Worth physician, has gone to Chicago and New York to take a post-graduate course. He will be gone a month or six weeks.

Dr. Sam F. Nave, of Kenedy, had the misfortune recently to break his arm while cranking an automobile.

Dr. Tom O. Staples, of Wylie, is at home after a thirty days' visit with friends and relatives in Kentucky.

Dr. C. P. Hines, of Dallas, was a visitor in Gainesville the 20th. He is a dentist, and will probably locate there.

Dr. C. M. Bishop, of Georgetown, is in Chicago attending the meeting of the Association of American Colleges.

Dr. S. T. R. Green, of Granbury, was recently stricken with broncho-pneumonia, but is now rapidly recovering.

Dr. H. S. Bunch, of Millsap, has purchased a residence in Weatherford, where he will move to practice his profession.

Dr. A. A. Bates and family, of Eagle Lake, left recently for Bronson, Kansas, where they expect to make their future home.

Dr. and Mrs. T. R. Sealy, of Santa Anna, have returned from Temple, where they have both been in the sanitarium for several weeks.

Dr. E. F. Gough, of Waxahachie, left January 22d for New York to take a special course in ear, eye, throat, nose and general surgery.

Dr. E. B. Fykes, of Fort Worth, charged with issuing prescriptions for narcotics, was found guilty by a county court jury and fined $50.

Dr. T. G. Bates, of Anna, Texas, will move his family to Lubbock as soon as he can get a home provided for them, and will engage in the practice of his profession.

Dr. J. S. Moulton and Miss Jean Vaughn, of Texarkana, were married in Dallas December 30th. They left for Texarkana. Dr. Moulton is connected with the Cotton Belt Hospital.

Dr. F. S. Craig, of Fort Worth, was fined $25 in the county court on a charge of illegally prescribing morphine and cocaine. Four other similar cases are pending against Dr. Craig.

Dr. M. M. Morrison, a practicing physician of Denison, has given up active practice in his profession and will interest himself in the organization of the American Health Insurance Company.

Dr. and Mrs. Auler, of Elgin, left recently for New Orleans, where they will spend awhile before going to Baltimore, at which place Dr. Auler will take up some special work in surgery and diseases of the kidney and bladder.

Dr. H. H. Smiley, of Dallas, has been named as chief surgeon for the Cotton Belt system, succeeding Dr. Charles A. Smith, who died a few days ago. Headquarters of Dr. Smiley will be the company's general hospital, Texarkana.

### The Factor of Poverty in Sanitation.

The factor of poverty in sanitary problems was discussed in Washington November 26th by Surgeon General William C. Gorgas, whose success in cleaning up Havana and the Panama Canal zone have brought him recognition as America's leading sanitarian. His audience was the Clinical Society of Surgeons, assembled in their twenty-fourth annual meeting. Dr. Gorgas said, in part:

"Such sanitary work as is necessary in the tropics is inexpensive, but measures directed against special disease are not the greatest good that can be accomplished by sanitation.

"Before these great results that we can all now see are possible for the sanitarian, we shall have to alleviate more or less the poverty at present existing in all civilized communities. Poverty is the greatest of all breeders of disease and the stone wall against which every sanitarian must finally impinge.

"During the last ten years of my sanitary work I have thought much on this subject. Of what practical measure could the modern sanitarian avail himself to alleviate the poverty of that class of our population which most needs sanitation? It is evident that this poverty is principally due to low wages; that low wages in modern communities are principally due to the fact that there are many more men competing for work than there are jobs to divide among these men. To alleviate this poverty two methods are possible, either a measure directed toward decreasing the number of men competing for jobs, or, on the other hand, measures directed toward increasing the number of jobs.

"The modern sanitarian can very easily decrease the number of men competing for jobs; if by next summer he should introduce infected stegomyia mosquitoes at a dozen different places in the Southern United States he could practically guarantee that when winter came we would have several million less persons competing for jobs in the United States than we have at present. This has been the method that man has been subject to for the last six or seven thousand years, but it does not appeal to me, nor, I believe, to yourselves. This method is at present being tried on a huge scale by means of the great war in Europe. I do not think that I risk much in predicting that, when this war is over and we shall have eliminated three or four million of the most vigorous workers in Europe, wages will rise and for a long time no man will be unable anywhere in Europe to get a job at pretty fair wages.

"But I am sure that every sanitarian would much rather adopt measures looking toward the increase of jobs rather than, as we have done in the past, submit to measures that decrease the number of competitors for jobs.

"I recently heard one of the members of the Cabinet state that in the United States 55 per cent of the arable land, for one reason or another, is being held out of use. Now suppose in the United States we could put into effect some measure that would force this 55 per cent of our arable land into use. The effect at once would be to double the number of jobs. If the jobs were doubled in number wages would be doubly increased. The only way I can think of forcing this unused land into use is a tax on land values.

"I therefore urge for your consideration, as the most important sanitary measure that can be at present devised, a tax on land values."

---

A Trying Time.—Juge—"Officer, what's the matter with the prisoner? Tell her to stop that crying—she's been at it fifteen minutes" (more sobs).

Officer—"Please, sir, I'm a-thinking she wants to be bailed out."—*Nebraska Awgwan.*

### Johnson County Physicians Pass Resolutions.

Recently the Physicians and Surgeons' Association of Johnson county met in a well attended session at the courthouse in Cleburne, and the following resolutions were unanimously adopted:

WHEREAS, There are certain persons who, after having received the benefit of the services of a physician and surgeon, refuse and fail, after a reasonable time has been given to pay their bills, though they are amply able to do so; and

WHEREAS, Such persons, in order to obtain the services of a physician and surgeon without paying for the same, refuse to pay their bills to one surgeon or physician and then go to another and obtain his services without paying their former medical bill; and

WHEREAS, It is but just and right that such persons should pay their overdue physician and surgeon bills; therefore, be it

*Resolved,* That it is the sense of this meeting that there should be a list of such persons who are able to pay, but refuse to pay their bills, made out and furnished to this Association; and be it further

*Resolved,* That no physician should desire to render his services for such a delinquent debtor until and unless such debtors shall either pay or satisfatorily secure such overdue indebtedness.

That this resolution is made and passed solely for the purpose of causing those who are justly indebted to members of this Association, and who are able to and will not pay such indebtedness to pay or secure such past due indebtedness; that this resolution is not passed for the purpose of causing any member of this Association to desist or fail to answer all charity or other calls, as has heretofore been the custom of the medical profession, but is passed solely for the purpose of assisting in the collection of indebtedness due the profession from those who are able and will not pay their just debts.

---

The broad social interests in life with which medicine is interwoven has called forth the enthusiasm and spirit of women in no uncertain way.—*Medical Review of Reviews.*

